# Supplementary material for: Prediction model for recommending coronary artery calcium score screening (CAC-prob) in cardiology outpatient units: A development study
Source: PLoS One. 2024 Sep 30;19(9):e0308890. doi: 10.1371/journal.pone.0308890 (PMC11441643; doi:10.1371/journal.pone.0308890)
Supplement: S1 File — (DOCX) [file pone.0308890.s001.docx]

**Table S1** Discriminative ability: Generalized ROC, Average ROC, Ordinal C-index

| **Discriminative ability** | **Average**  **Value** | **0 vs >0** | **<100 vs ≥100** | **0 vs 1-99** | **0 vs ≥100** | **1-99 vs ≥100** |
| --- | --- | --- | --- | --- | --- | --- |
| Generalized ROC | 0.73 |  |  | 0.75 | 0.83 | 0.60 |
| Average ROC | 0.76 | 0.79 | 0.73 |  |  |  |
| Ordinal C-index | 0.81 |  |  |  |  |  |

**Table S2** Risk classification table for each lower pairs cut point based on the estimated probability of CAC >0

| **Cut point** | **number of classified patients** | **CAC =0**  **(n=107)** | **CAC 1-99**  **(n=85)** | **CAC ≥100**  **(n=95)** | **CAC ≥400**  **(n=39)** | **correct classified**  **rate** |
| --- | --- | --- | --- | --- | --- | --- |
| ≥0.45 | 217 | 54 (50%) | 73 (86%) | 90 (95%) | 36 (92%) | 77% |
| <0.45 | 70 | 53 (50%) | 12 (14%) | 5 (5%) | 3 (8%) |  |
| ≥0.50 | 203 | 45 (42%) | 69 (81%) | 89 (94%) | 36 (92%) | 78% |
| <0.50 | 84 | 62 (58%) | 16 (19%) | 6 (6%) | 3 (8%) |  |
| ≥0.55 | 179 | 34 (32%) | 62 (73%) | 83 (85%) | 33 (85%) | 75% |
| <0.55 | 108 | 73 (68%) | 23 (27%) | 12 (15%) | 6 (15%) |  |

*Total of 287 patients used for calculating the probability were based on unimputed data set.

| **Cut point** | **number of classified patients** | **CAC =0**  **(n=54)** | **CAC 1-99**  **(n=73)** | **CAC ≥100**  **(n=90)** | **CAC ≥400**  **(n=36)** | **correct classified**  **rate** |
| --- | --- | --- | --- | --- | --- | --- |
| ≥0.20 | 177 | 34 (63%) | 62 (85%) | 81 (90%) | 32 (89%) | 47% |
| <0.20 | 40 | 20 (37%) | 11 (15%) | 9 (10%) | 4 (11%) |  |
| ≥0.25 | 146 | 22 (41%) | 54 (74%) | 70 (78%) | 30 (83%) | 54% |
| <0.25 | 71 | 32 (59%) | 19 (26%) | 20 (22%) | 6 (17%) |  |
| ≥0.30 | 122 | 19 (35%) | 45 (62%) | 58 (64%) | 27 (75%) | 56% |
| <0.30 | 95 | 35 (65%) | 28 (38%) | 32 (36%) | 9 (25%) |  |

**Table S3** Risk classification table for each cut point based on the estimated probability of CAC ≥100, when 0.45 was set as the lower pairs cut point to determine low risk of CAC >0

*Total of 287 patients used for calculating the probability were based on unimputed data set.

| **Cut point** | **number of classified patients** | **CAC =0**  **(n=45)** | **CAC 1-99**  **(n=69)** | **CAC ≥100**  **(n=89)** | **CAC ≥400**  **(n=36)** | **correct classified**  **rate** |
| --- | --- | --- | --- | --- | --- | --- |
| ≥0.20 | 177 | 34 (76%) | 62 (90%) | 81 (91%) | 32 (89%) | 41% |
| <0.20 | 26 | 11 (24%) | 7 (10%) | 8 (9%) | 4 (11%) |  |
| ≥0.25 | 146 | 22 (49%) | 54 (78%) | 70 (79%) | 30 (83%) | 51% |
| <0.25 | 57 | 23 (51%) | 15 (22%) | 19 (21%) | 6 (17%) |  |
| ≥0.30 | 122 | 19 (42%) | 45 (65%) | 58 (65%) | 27 (75%) | 53% |
| <0.30 | 81 | 26 (58%) | 24 (35%) | 31 (35%) | 9 (25%) |  |

**Table S4** Risk classification table for each cut point based on the estimated probability of CAC ≥100, when 0.50 was set as the lower pairs cut point to determine low risk of CAC >0

* Total of 287 patients used for calculating the probability were based on unimputed data set.

| **Cut point** | **number of classified patients** | **CAC =0**  **(n=34)** | **CAC 1-99**  **(n=62)** | **CAC ≥100**  **(n=83)** | **CAC ≥400**  **(n=36)** | **correct classified**  **rate** |
| --- | --- | --- | --- | --- | --- | --- |
| ≥0.20 | 177 | 34 (100%) | 62 (100%) | 81 (98%%) | 32 (97%) | 32% |
| <0.20 | 2 | 0 (0%) | 0 (0%) | 2 (2%) | 1 (3%) |  |
| ≥0.25 | 146 | 22 (65%) | 54 (87%) | 70 (84%) | 30 (91%) | 44% |
| <0.25 | 33 | 12 (35%) | 8 (13%) | 13 (16%) | 3 (9%) |  |
| ≥0.30 | 122 | 19 (56%) | 45 (73%) | 58 (70%) | 27 (82%) | 47% |
| <0.30 | 57 | 15 (44% | 17 (27%) | 25 (30%) | 6 (18%) |  |

**Table S5** Risk classification table for each cut point based on the estimated probability of CAC ≥100, when 0.55 was set as the lower pairs cut point to determine low risk of CAC >0

* Total of 287 patients used for calculating the probability were based on unimputed data set.
